# Trajectory of hiPSCs derived neural progenitor cells differentiation into dermal papilla-like cells and their characteristics

**DOI:** 10.1038/s41598-023-40398-w

**Published:** 2023-08-30

**Authors:** Andrei Riabinin, Ekaterina Kalabusheva, Anastasia Khrustaleva, Mikhail Akulinin, Alexander Tyakht, Egor Osidak, Elina Chermnykh, Andrey Vasiliev, Ekaterina Vorotelyak

**Affiliations:** 1 Koltzov Institute of Developmental Biology of Russian Academy of Sciences, Moscow, Russia; 2 Institute of Gene Biology of Russian Academy of Sciences, Moscow, Russia; 3 Imtek LTD, Moscow, Russia

**Keywords:** Differentiation, Embryology, Stem cells

## Abstract

Dermal papilla cells (DPCs) play roles in key functions of the epidermis such as hair generation. The use of human induced pluripotent cells (hiPSCs) makes it possible to obtain DP-like cells and study the molecular mechanisms of DPC development during embryogenesis. In this work, we studied the phenotypic trajectory of hiPSCs during their differentiation into DP-like cells and evaluated the epithelial-mesenchymal interaction potential of the resulting cell line. Specifically, we differentiated hiPSCs into neural progenitor cells (NPCs) and subsequently into DP-like cells. Analysis of bulk RNA-seq data during this process enabled us to observe gene expression dynamics during five stages of dermal differentiation. Furthermore, functional assays (organoids in both collagen gels and hanging drop cultures and tubulogenesis assays) revealed that the dermal cell lines we generated could interact with epidermal cells.

## Introduction

Hair follicle (HFs) are skin derivatives involved in skin development and homeostasis. Among the skin derivatives that can be generated in the laboratory, HFs have yielded the most successful results. In mammals, HFs are responsible for the generation, growth, and development of hair shafts, which, in turn, provide thermoregulation, sensory information, and protection from environmental influences, and are important for social communication^1,2^. Dermal papilla cells (DPCs) are located at the bases of HFs where they control the development and cycling of HF cells and are involved in HF homeostasis^3^.

The origin of DPCs during human embryogenesis differs depending on the anatomic site in question. Cranial DPCs and facial HFs originate from neural crest cells (NCCs), while dorsal and ventral trunk DPCs develop from somitic and lateral plate dermomyotome^4^. However, due to bioethical limitations, the process of NCC differentiation into DPCs, as well as intermediate stages of differentiation in human skin morphogenesis, are not well understood^4^.

An important instrument in modern developmental biology, regenerative medicine and genetic disease modelling is hiPSCs^5,6^. To date, several protocols have been developed that facilitate the direct differentiation of human induced pluripotent cells (hiPSCs) into DPCs^7–9^. Recent studies have also reported the generation of autologous two-component living skin equivalents (LSEs) from hiPSC-derived fibroblasts and KCs^10,11^. However, despite their broad differentiation protocol applicability, the trajectory of hiPSCs into facial HFs differentiation and role of epithelial-mesenchymal interaction in these processes remains not fully understood.

In this study, we obtained DP-like cells from hiPSC-derived neural progenitor cells (hiPSC-NPCs), which are direct NCC analogs in culture, and validated their phenotypes. To better understand the intermediate stages and underlying mechanisms of differentiation, we used bulk RNA-seq to investigate the expression dynamics of neural and fibroblast markers at five time points during the differentiation of hiPSCs into DP-like cells. To study epithelial-mesenchymal interactions during hiPSC-DPC differentiation, cells at different stages of differentiation were co-cultured with primary human KCs and the resulting conditioned medium was used in tubulogenesis assays^12^. Finally, we explored the potential of our DP-like cells to generate three-dimensional systems such as LSEs and organoids, mimicking the early stages of folliculogenesis in vitro.

## Materials and methods

### Cell culture

Human KYOU-DXR0109B hiPSCs [201B7] were used for differentiation between passages 15 and 30 (ACS-1023, ATCC, Tokyo, Japan). Human DPCs (hDPCs; used between passages 0 and 3) and human KCs (hKCs; used between passages 0 and 1) obtained from primary skin cultures from 3 independent donors were used as positive controls in each experiment.

### Mice

This study was approved by the Local Ethical Committee for Clinical Research of the Pirogov Russian National Research Medical University (Protocol No. 9, 2019). NOD/SCID mice (age: 10–12 weeks; weight: 25 g; n = 4/group) were obtained from Charles River and housed under the following specific pathogen-free conditions: temperature: 22 ± 3 °C, relative humidity: 40–60%, and a 12-h light/dark cycle.

All animals had free access to water and standard compound feed. The animals were allowed to acclimate to their environments for 2 weeks prior to surgery. Surgeries were performed under general anesthesia. The animals were euthanized with a three-fold overdose of 2,2,2-tribromoethanol (Sigma-Aldrich). All methods were carried out in accordance with relevant guidelines and regulations.

All methods are reported in accordance with ARRIVE guidelines (https://arriveguidelines.org).

### Cultivation of hiPSCs

hiPSCs were cultured for 2 weeks according to the manufacturer’s recommendations before being subjected to differentiation in mTeSR1 medium (Stemcell, Vancouver, Canada). The medium was replaced daily and cells were maintained in a multi-gas incubator (in an atmosphere of 5% oxygen and 5% carbon dioxide).

### Differentiation of hiPSCs into hiPSC-NPCs and cultivation of hiPSC-NPCs

Differentiation of hiPSCs into hiPSC-NPCs was performed over a 6-week period and cells were cultivated in a multi-gas incubator (in an atmosphere of 5% oxygen and 5% carbon dioxide) using PSC neural induction medium (Gibco, Uoltem, USA) with 1mM penicillin/streptomycin (Gibco) and 1 mM glutamine (Gibco) according to the manufacturer’s instructions. The hiPSC-NPCs were passaged using accutase (Gibco, Waltham, Massachusetts, U.S.A.) (3 mg/ml) with a confluence of 70%. HiPSC-NPCs seeding density after passage was 40000 cells/cm^2^.

### Differentiation of hiPSC-NPCs into hiPSC-DPCs

hiPSC-NPCs were passaged on plastic using accutase (3 mg/ml) according to standard protocols and immediately transferred to differentiation medium: DMEM/F12 (PanEco, Moscow, Russia) (glucose concentration 4,5 mg/ml) supplemented with 10% fetal bovine serum (FBS), 1 mM L-glutamine (Gibco), 1x insulin-transferrin-selenium (Gibco), and 1 mM penicillin/streptomycin (Gibco). Subsequently, the medium was changed every 3 days for 8 weeks. hiPSC-DPCs were then cultured in AmnioMAX-II medium (Gibco). The reason of switch from DMEM/F12 medium with 10% FBS, Glutamin and P/S to AmnioMAX-II was the end of differentiation and better cultivation condition for hiPSCs-DPCs on this medium with low FBS percentage. Passaging during differentiation and cultivation was performed with 0.05% trypsin-EDTA (Gibco) and cells were maintained in an atmosphere of 21% oxygen and 5% carbon dioxide. Cells seeding density after passage in all cases was 40000 cells/cm^2^. HiPSC-DPCs were used for all further experiments at passages 1–15.

### Isolation of hDPCs and hKCs from human skin

#### Material pretreatment

Human scalp skin, kindly provided by P. A. Herzen Moscow research oncological institute with the informed consent of donors, was used as a source of human adult dermal and epidermal cells. The skin was first washed in Hank’s solution (PanEco) supplemented with 1.2% gentamicin. Subsequently, hair was removed from the skin to prevent contamination.

#### hKCs isolation

Skin fragments were removed from the dermis with a scalpel as close to the basement membrane as possible. To isolate KCs, the skin was incubated with 2 μg/ml dispase at 4 °C, and the epidermal layer was separated from the dermis and transferred to a 10 ml of 0.025% trypsin solution (Gibco) diluted in Dulbecco’s phosphate-buffered saline (DPBS) (PanEco) for 25 min at 37 °C. The suspension was then transferred to another 15-ml tube, 500 μl of FBS were added to inhibit trypsin activity, and the tube was centrifuged for 10 min at 400 × RCF. Before plating KCs, the culture dishes were treated with a solution of collagen I (Sigma) to promote cell adhesion and incubated for 10 min at 37 °C. After centrifugation, the cell pellet was diluted in KC medium composed of DMEM/F12 (PanEco) (glucose concentration 4,5 mg/ml) supplemented with 10 ng/ml epidermal growth factor (EGF) (Gibco), 5 μg/ml insulin (Sigma), 10^–6^ M isoproterenol (Sigma), 10% FBS, 1 mM glutamine, and 1 mM penicillin/streptomycin (all from Gibco). 25 cm^2^ flasks were coated with collagen I after 30 min of incubation with 1 mg/ml collagen I solution (Sigma) (1 ml per 5 cm^2^). hKCs were cultured on collagen I coated flasks in CNT-07 medium (CellNTec, Bern, Switzerland). Cultivation of all hKCs in other experiments was performed on the same coating. Medium was changed every two days, and cells were maintained in an incubator with 21% oxygen and 5% carbon dioxide.

#### hDPCs isolation

Scalp skin was cut into 1–2-cm wide strips and transferred to DMEM (glucose concentration 4,5 mg/ml) (PanEco) with 1 mM glutamine (Gibco), 0.9% gentamicin (Sigma), and 0.5% dispase (Gibco). The skin was then placed at 4 °C and incubated overnight. The next day, subcutaneous fat and HFs were manually removed from the skin strips and were incubated for 2 h at 37 °C in DMEM with 0.2% type I collagenase (Gibco) and centrifuged for 10 min at 400 × RCF. The material was incubated in fresh 0.2% type I collagenase (Gibco) in DMEM for 4–5 h, centrifuged for 7 min at 30 × RCF, and then washed several times in Hank’s solution (PanEco) with centrifugation for 5 min at 30 × RCF to remove single cells. The resulted precipitate consists only from hair follicles bulbs and dermal papilla. Isolated DPCs were transferred to culture dishes and maintained in AmnioMAX-II medium (Gibco), which was changed every 3 days. Passaging was performed as described above for hiPSC-DPCs. Cells were cultivated under conditions of 21% oxygen and 5% carbon dioxide.

### Lentiviral transfection of hDPCs and hiPSC-DPCs

HDPCs and hiPSC-DPCs were incubated with red fluorescent protein (RFP)-tagged (TagRFP) viral particles (Eurogen, Moscow, Russia) in tenfold excess relative to the concentration of cells in AmnioMAX-II medium (Gibco) supplemented with 10 µg/ml polybrene (Sigma).

### Formation of organoids mimicking the early stages of folliculogenesis

To generate organoids, the following cell lines were used: hKCs (1–2 passages) cultured in DMEM/F12 (glucose concentration 4,5 mg/ml) supplemented with 10% FBS (Gibco), 5 μg/ml insulin (Sigma), 10 ng/ml EGF (Gibco), and 10^–6^ M isoproterenol (Gibco); hKCs (1 passage) cultivated in CNT-07 medium (CellNTec); and hiPSC-DPCs cultured in DMEM/F12 with 10% FBS and 1 mM glutamine (Gibco). To generate organoids, the following combinations of cell types were used: hDPCs + hKCs (cultivated in different media) and RFP-labeled hiPSC-DPCs + hKCs.

To generate organoids with only one cell line, 5000 epidermal or 5000 dermal cells were used. In co-cultures with epidermal and dermal components, 2500 cells from each line were used. Organoids were generated via the hanging drop method. Briefly, the cells were passaged with 0.05% trypsin (Gibco) and placed on the lid of a Petri dish in a 20-μl drop of DMEM/F12) (glucose concentration 4,5 mg/ml) (PanEco) with 10% FBS (Gibco). The lid was then turned over and placed on a 96-well plate with PBS (PanEco) dispensed in the bottom of the wells. Organoid maturation took 3 days, after which they were fixed with freshly prepared 4% PFA solution (Sigma) for 1 h. The fixative was then removed by washing, and organoids were transferred to a fresh plate and incubated in a primary antibody solution with the recommended by manufacturer concentrations containing 5% BSA (Sigma), 1% Triton X-100 (Sigma), 1% Tween 20 (Sigma), and several sodium azide crystals overnight at 4 °C. The primary antibodies were then removed by washing and the secondary antibodies (dilution 1: 500) were added to the block solution and incubated overnight at 4 °C. The next day, the secondary antibodies were removed by washing and the nuclei were stained with 10 μM Draq5 (Gibco) diluted in PBS (PanEco). The cells were then passed through ascending concentrations of fructose solutions. Finally, the organoids were placed on glass slides with spacers in the form of coverslip fragments and covered with coverslips. Images were taken with a Zeiss (Jena, Germany) LSM T-PMT confocal microscope.

### Co-cultivation of hiPSC-DPCs with hKCs during differentiation

During differentiation, iPSC-DPCs were cultured in polyester membrane cell culture inserts with 8-μm pores in 24-well plates (Corning, Corning, USA). In these experiments, we studied the effect of co-cultivation on dermal cells during differentiation and on hKCs. In the first experiment, hKCs were plated in inserts (Corning) (100,000 cells per transwell), whereas hiPSC-NPCs (negative differentiation control), hiPSC-DPCs at 2, 4, 6, or 8 weeks of differentiation, or hDPCs (100,000 cells per well) were plated on the bottom of the 24-well plate. In the second experiment, hKCs were seeded on the bottom of the wells and the remaining aforementioned cell types were plated in the inserts. For each experimental group, there was also a negative control without co-cultivation. Twenty-four hours before co-cultivation, the relevant cell types were plated on either inserts or 24-well plates and cultured separately in hiPSC-DPCs differentiation medium (DMEM/F12 [PanEco] (glucose concentration 4,5 mg/ml) (protocol of hiPSC-DPCs was described above) supplemented with 10% FBS [#10270], 1 mM L-glutamine (Gibco), 1 × insulin-transferrin-selenium, and 1 mM penicillin/streptomycin [Gibco]). In each group, co-cultivation was performed for 3 days in hiPSC-DPC medium, after which the inserts were discarded and the cells in the 24-well plate were fixed and used for the immunocytochemical detection of α-SMA, β-tubulin III, GFAP, versican, vimentin, and KI67 (Table 1). Images were taken with an Olympus IX73 microscope using an Olympus DP74 digital color camera. KI67^+^ cells were manually counted in ImageJ.

**Table 1:** List of the first antibodies used for immunocytochemical and immunohistochemical detection of markers.

Antigen	Catalog number	Producer	Dilution	Type and host
Versican	AF3054	R&D systems	1: 20	Polyclonal lgG, goat
Vimentin	Ab8988	Abcam	1: 50	Monoclonal lgG, mouse [54B3]
Fibronektin	Ab2413	Abcam	1: 200	Polyclonal lgG, rabbit
S100A4	Ab27957	Abcam	1: 50	Polyclonal lgG, rabbit
αSMA	Ab5694	Abcam	1: 100	Polyclonal lgG, rabbit
К14	Ab181595	Abcam	1: 500	Monoclonal lgG, rabbit [EPR17350]
K6	Ab52620	Abcam	1: 250	Monoclonal lgG, rabbit [EPR1603Y]
GFAP	Ab16667	Abcam	1: 250	Monoclonal lgG, rabbit
Ki67	MAB-5628	Sigma	1: 200	Monoclonal lgG, mouse
β-3-tubulin	Ab151318	Abcam	1: 500	Monoclonal lgG, rabbit

#### Generation of LSEs with organoids

LSEs were generated based on the protocol reported by Abaci et al.^13^. LSEs were fabricated in 3 stages: gel fabrication, cell incorporation, and cultivation. First, a 50 mg/ml high-density collagen I gel (Viscoll; lyophilized sterile atelocollagen from porcine tendons [Imtek Ltd., Moscow, Russia]) was prepared. The resulting gel was poured into silicone molds with wells. The gel was then polymerized for 1 h at 37 °C. The resulting polymerized gels were then transferred to 12-well plates and 1.8 million hDPCs or hiPSC-DPCs (after 6 weeks of differentiation) in 100 μl of DMEM (PanEco) (glucose concentration 4,5 mg/ml) with 10% FBS, 1 × penicillin/streptomycin, 1 × Glutamax, and 1 × ascorbic acid (all from Gibco) were added. In the negative control group, DPCs were not added. Immediately after plating, the gels were centrifuged at 10 × g for 5 min to settle the cells in the bottoms of the wells. Next, 1.3 ml of medium was carefully added. 2.5 million hKCs in CNT-PR-Air lift medium (CellNTEC) were seeded onto the gels. The gels were then transferred into 6-well plates with 3 ml of CNT-PR-Air lift medium (Thermo Fisher Scientific). LSEs were cultured for 10 days and the medium was changed every other day.

#### Tubulogenesis assay

The following cell mixes were used: medium without conditioned medium (negative control); medium (DMEM/F12 [PanEco] (glucose concentration 4,5 mg/ml) with 10% FBS, Glutamax, insulin-transferrin-selenium, and EGF [all from Gibco]) with 10% conditioned medium from hiPSC-DPCs at different stages of differentiation (0–4, 12–16, 24–28, and 40–42 days of differentiation); and conditioned medium from hDPCs (positive control). Three wells were used for each group. A 10 mg/ml collagen solution was first poured into the wells of a 24-well plate (500 μl per well) and allowed to polymerize at 37 °C. After 1 h, 3D-printed 6 × 6 ABS (Acroline Butadiene Styrene) cylinders were placed on top of the polymerized gels. Next, a 10 mg/ml collagen gel solution was poured around the cylinder (700 μl per well). Thereafter, polymerization was carried out as described above and the cylinders were removed from the wells. Primary hKCs were seeded in the resulting “pocket” (500,000 hKCs per well) in a minimal amount (50–100 μl) of medium (DMEM/F12 [PanEco] with 10% FBS [Gibco], 5 μg/ml insulin [Sigma], 10 ng/ml EGF [Gibco], and 10^–6^ M isoproterenol [Gibco]). One hour after seeding, another 500 µl of the relevant medium were added to each well. The medium was changed every other day for 14 days. After 14 days, the cells were stained with Hoechst (Sigma) and images of the resulting tubules were acquired with an inverted fluorescence microscope (IX51, Olympus, Tokyo, Japan) and Olympus DP70 camera. All measurements were acquired in triplicate and the lengths and numbers of tubules in each image were determined manually.

#### hiPSC-DPC tumorigenesis assay

Mice (detailed information was described above) were anesthetized via intraperitoneal administration of 2,2,2,-tribromoethanol (Sigma) (250 mg/kg body weight). A 1-cm incision was made through the skin and peritoneum of the middle of the abdomen, approximately 1 cm from the preputial glands. The testes and adipose tissue were retracted with forceps. Using an insulin syringe, an incision was made on the testicular capsule. Thereafter, a suspension of 1 × 10^6^ iPSC-DPCs was injected under the capsule of the right testis with a Hamilton syringe, while a suspension of 1 × 10^6^ iPSCs was injected under the capsule of the left testis. The testes were then returned to the abdominal cavity, after which two sutures were applied to the peritoneum and skin (Sofsilk 5–0 silk thread, 16-mm needle). Four months after transplantation, the animals were euthanized with a threefold overdose of 2,2,2,-tribromoethanol. The fragments of the left testis were cut into 1 × 1-cm pieces and embedded in OCT medium (Thermo Fisher Scientific) for cryoblock preparation. The resulting blocks were stored at − 80 °C for 2 hours and then transferred to − 20 °C. Cryoblocks were used to prepare with Thermofisher HM520 frozen Sects. (7 µm), which were mounted on polylysine-coated slides. All methods were carried out in accordance with relevant guidelines and regulations.

All methods are reported in accordance with ARRIVE guidelines (https://arriveguidelines.org).

#### Histology

Tumor sections were fixed in 10% formalin for 10 min and stained with hematoxylin and eosin (BioVitrum, Moscow, Russia). Images were acquired with an Olympus IX73 microscope using an Olympus DP74 digital color camera.

#### Immunocytochemistry

Cells were washed with PBS (PanEco) and fixed in 4% PFA/PBS, pH 7.2 (Sigma). The cells were washed in PBS (PanEco) three times for 5 min each, and primary antibodies were added in blocking solution (Table 1) (0.1% Triton X-100 diluted in 5% BSA with several sodium azide crystals [all from Sigma]). Incubation was performed overnight at 4 °C. Cells were then washed four times for 15 min each and the corresponding secondary antibodies (Table 2) (1:500) were added; incubation was performed for 1 h in the dark at room temperature. Cells were then washed with PBS (PanEco) three times for 5 min each in the dark. Subsequently, cells were stained with DAPI (Sigma; 1:3000). Images were acquired with an Olympus IX73 microscope and an Olympus DP74 digital color camera.

**Table 2:** List of second antibodies used for immunocytochemical and immunohistochemical detection of markers.

Antigen	Catalog number	Producer	Dilution	Type and *fluorochrome*
lgG (H + L), rabbit	A32731	Invitrogen	1: 500	Alexa 488 + goat polyclonal lgG
lgG (H + L), rabbit	A11035	Invitrogen	1: 500	Alexa 546 + goat polyclonal lgG
lgG (H + L), mouse	A21002	Invitrogen	1: 500	Alexa 488 + donkey polyclonal lgG
lgG (H + L,) mouse	A32727	Invitrogen	1: 500	Alexa 555 + goat polyclonal lgG
lgG (H + L), goat	A11055	Invitrogen	1: 500	Alexa 488 + donkey polyclonal lgG

#### Immunohistochemistry

LSEs with organoids were fixed for 1 h at room temperature in freshly prepared 4% PFA and serially transferred into 30% and 60% sucrose solutions. Thereafter, the samples were embedded in OCT (Gibco), frozen in cryoblocks, and stored at − 80 °C. Frozen LSE Sects. (10 µm) were prepared with Thermofisher HM520, transferred to polylysine-coated slides, and dried at room temperature for one day. For staining, primary antibodies (Table 1) were diluted in block solution (0.5% Triton X-100 in 5% BSA [Sigma] diluted in PBS [PanEco] with several sodium azide crystals; 100 μl per section) and the samples were incubated overnight at 4 °C. Subsequently, the samples were washed three times with PBS for 5 min each. A solution of the corresponding secondary antibodies (Table 2) (100 μl per section) with DAPI (1:500; Sigma) was prepared and incubated with the samples for 1 h at room temperature in the dark. Finally, the samples were washed three times for 5 min each with PBS (PanEco), the remaining liquid was removed from the slides, and specimens were embedded in 1% glycerol in PBS and covered with fat-free 200-μm coverslips. Images of LSEs were acquired with an Olympus IX73 microscope with an Olympus DP74 camera.

#### Real-time PCR

RNA was isolated using RNAzol (Sigma) and purified by sequential centrifugation with solutions of ethanol and isopropanol. Genomic DNA elimination and reverse transcription were performed with the QuantiTect Reverse Transcription kit (Thermo Fisher Scientific) in accordance with the manufacturer’s protocol. PCR solutions were prepared with the high-sensitivity SYBR Low-ROX kit (Eurogen, Russia) according to standard protocols (list of primers in Table 3). Reactions were run on a CFX96 Real-Time PCR Detection System (BioRad, Berkeley, USA). Amplification program: 95 °C for 5 min; 45 cycles (95 °C for 20 s; 60 °C for 20 s; 72 °C for 30 s + plate read). Human DNA isolated from HaCaT cells (100 mM) was used to standardize PCR results. Differential expression values were obtained from 3 independent samples per group. The real-time PCR data were normalized on GAPDH and HPRT expession.

**Table 3:** List of Quantitative-PCR primers used in this study.

Gene	Forward primer	Reverse primer
VCAN	CAGCTCTTTGCTGCCTATGA	CTCCTGCCTTTCCCATCTTA
Vimentin	TACAGGAAGCTGCTGGAAGG	ACCAGAGGGAGTGAATCCAG
ACTA2	AGCCAAGCACTGTCAGGAAT	CCAGAGCCATTGTCACACAC
Fibronectin 1	GACGCATCACTTGCACTTCT	GCAGGTTTCCTGATTATCCT
Nestin	TGTGGCAAAGGAGCCTACTC	ATGGAGCAGGCAAGAGATTC
TUBB3	AAAAGGCTTCACAAGGGAAAGG	TCCGAGTCGCCCACGTAGTT
CD133	AATCTCCCTGTTTGGTGATTT	TTTTCTGTTTGGTGGGTTTT
HPRT	ACCAGGTTATGACCTTGATT	AAGTTGGCCTAGTTTATGTT
GAPDH	CGTAGCTAGGCCTCAAGAC	GCTGCGGGCTAATCAATTTATAG
NANOG	TGTCCAAGAGAAAGCATAAGAAAA	TGGAGGCCAAAATAGGAAGA
SOX2	TTGCTGCCTCTTTAAGACTAGGA	TAAGCCTGGGGCTCAAACT

#### Bulk RNA-seq

RNA was extracted from samples (hiPSC-NPCs, hiPSC-DPCs after 2, 4, 6, and 8 weeks of differentiation, and hDPCs) using the PureLink RNA Mini Kit (Thermo Fisher Scientific) according to the manufacturer’s recommendations. RNA samples were analyzed by Genetico (https://genetico.ru/).

#### Quantitative fluorescence intensity statistics

The images were analyzed in ImageJ v.1.53 K software. The area of β-tubulin III expression were measured by using threshold algorithm after 8-bit images conversion. The intensity of fluorescence of fibronectin, vimentin, αSMA and versican were measured after 8-bit images conversion.

#### Bioinformatics

Short read quality was checked with FastQC software^14^. Adaptor trimming was performed with Cutadapt (23); sequence ends with quality scores < 20 were trimmed and sequences of < 30 base pairs were removed using Sickle^15^. The trimmed reads were mapped to GRCh38 (hg38) and the number of reads per gene was counted with STAR^16^. Differential expression analysis was conducted using the edgeR package^17^. Row counts were filtered to include those that had greater than 1 count per million (CPM > 1) in at least two replicate samples. The remaining read counts were then normalized using the trimmed mean of values method (TMM) implemented in edgeR to account for differences in library size. Generalized linear models (GLMs) and the likelihood ratio test in edgeR were used to identify differentially expressed genes (DEGs) and the Benjamini–Hochberg false-discovery rate correction was applied (alpha = 0.05). Gene ontology (GO) enrichment and Reactome pathway analyses of the DEGs were performed using the WebGestaltR package^18^. The ggplot2 and heatmap.2 R packages were used for data visualization.

#### Statistical analysis

Statistical analysis of bioinformatic data is described in the supplemental information file. Statistical analyses in all other experiments were performed using STATISTIKA (v.10.0). The Mann–Whitney U test was used to compare qPCR, KI67 positivity, and tubulogenesis assay data between groups. Results with *p* values < 0.05 were considered significantly different from the null hypothesis.

#### Significance statement

We showed for the first time that human NPCs can differentiate into bipotent progenitors that give rise to Schwann cells, autonomic neurons, facial skull cartilages, DPCs, and other mesenchymal cell subpopulations. We also obtained a DP-like cell line, analyzed the intermediate stages of this differentiation and demonstrated their functional potential in epithelia-mesenchymal interaction in vitro.

## Results and discussion

### Generation of hiPSC-DPCs from hiPSC-NPCs

The differentiation of iPSC-NPCs into DPCs was induced by a non-specific differentiation factor: FBS. Despite its low specificity and non-standardized composition, several reports have demonstrated that FBS can be used to differentiate neural stem cells^7,19^. Herein, stepwise dynamics in hiPSC-DPC morphology and marker expression during differentiation, as well as their functional potential after differentiation, were analyzed.

**Figure 1:**
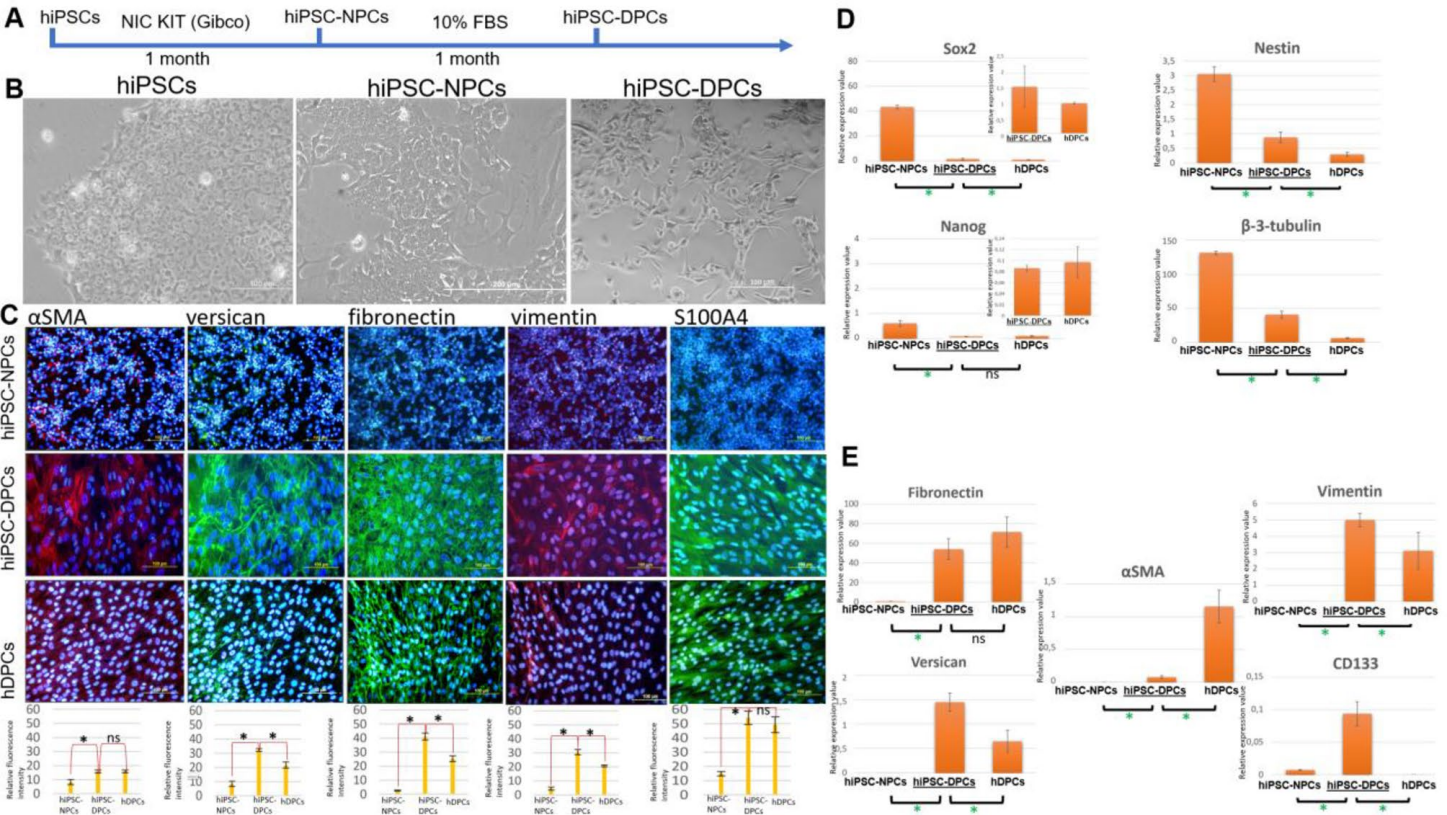
Morphology and marker expression of hiPSC-DPCs. (**A**) Schematic showing differentiation of hiPSCs into hiPSC-DPCs. (**B**) iPSCs at different stages of differentiation into DP-like cells. 1A: Phase contrast. Scale bar: 200 μm in the left and right images and 400 μm in the center. (**C**) Immunofluorescent detection of DPC markers (versican, αSMA, S100A4, fibronectin, and vimentin) in hiPSC-NPCs (negative control), hiPSC-DPCs (experimental group), and hDPCs (positive control). Scale bar: 100 and 200 μm. Below: versican, αSMA, S100A4, fibronectin, and vimentin fluorescence intensity in hiPSC-NPCs, hiPSC-DPCs and hDPCs. Scale is in relative value; *p ≤ 0.05; ns—non significane; p ≥ 0.05, Mann–Whitney test. (**D**) qPCR-based expression levels of pluripotency (Sox2, Nanog) and NPC (Sox2, Nestin, β-tubulin III) markers in hiPSCs (negative control 1), hiPSC-NPCs (negative control 2), hiPSC-DPCs (experimental group), and hDPCs (positive control). *p ≤ 0.05; ns—non significane; p ≥ 0.05, Mann–Whitney test. (**E**) qPCR-based expression levels of the fibroblast markers fibronectin, vimentin, versican, αSMA, and CD133 in hiPSCs (negative control 1), hiPSC-NPCs (negative control 2), hiPSC-DPCs (experimental group), and hDPCs (positive control). *p ≤ 0.05; ns—non significane; *p* ≥ 0.05, Mann–Whitney test.

hiPSC-NPCs obtained from hiPSCs were differentiated into hiPSC-DPCs as previously described^7^ (Fig. 1A). The resulting cells exhibited a fibroblast-like morphology (small, flat, spindle-shaped cells) and tended to form clumps in culture (Fig. 1B). The cells also expressed the fibroblast markers versican, αSMA, S100A4, fibronectin, vimentin, and CD133 (Fig. 1C,E).

Compared to the positive control (hDPCs), the hiPSC-DPC line showed higher levels of VCAN and vimentin expression, similar levels of fibronectin and S100A4 expression (Fig. 1C,E). Additionally, unlike the hDPCs, the hiPSC-DPC line expressed CD133 (Fig. 1E). This fact demonstrates us, that hiPSC-DPCs have some differences in mesenchymal markers expression as compared to donor adult dermal papilla cells. Successful differentiation was also demonstrated by the lack of pluripotency (SOX2 and NANOG) and NPC (Sox2, Nestin, TUBB3) marker expression in the hiPSC-DPC line compared to the negative controls (Fig. 1D), as well as the negative result from the tumorigenesis test (Figure S1A–C)^20^. The formation of teratomas from hiPSCs proved that they can differentiate into 3 germ layers and was used as positive control. The absence of malignant growth after hiPSC-DPCs transplantation proved, that there were no undifferentiated hiPSCs in the hiPSC-DPCs line (Figure S1A-C).

After prolonged cultivation (more than 10 passages), the hiPSC-DPCs cells demonstrated active proliferation. A distinguishing feature of this cell line was the formation of small, multilayered clusters and a lack of a confluent monolayer (primary adult DPCs from adult donors proliferate in a monolayer). This can simulate the process of cell aggregation during embryonic skin DPC formation. This fact can also be explained by the fact that newly differentiated hiPSC-DPCs are closer to embryonic DPCs than to adult ones.

Taken together, these characteristics suggest that our method of producing hiPSC-DPCs is reproducible and robust. The phenotype and expression of DPC markers in the resulting cells are consistent with published data ^7^.

### Analysis of phenotype dynamics during differentiation

**Figure 2:**
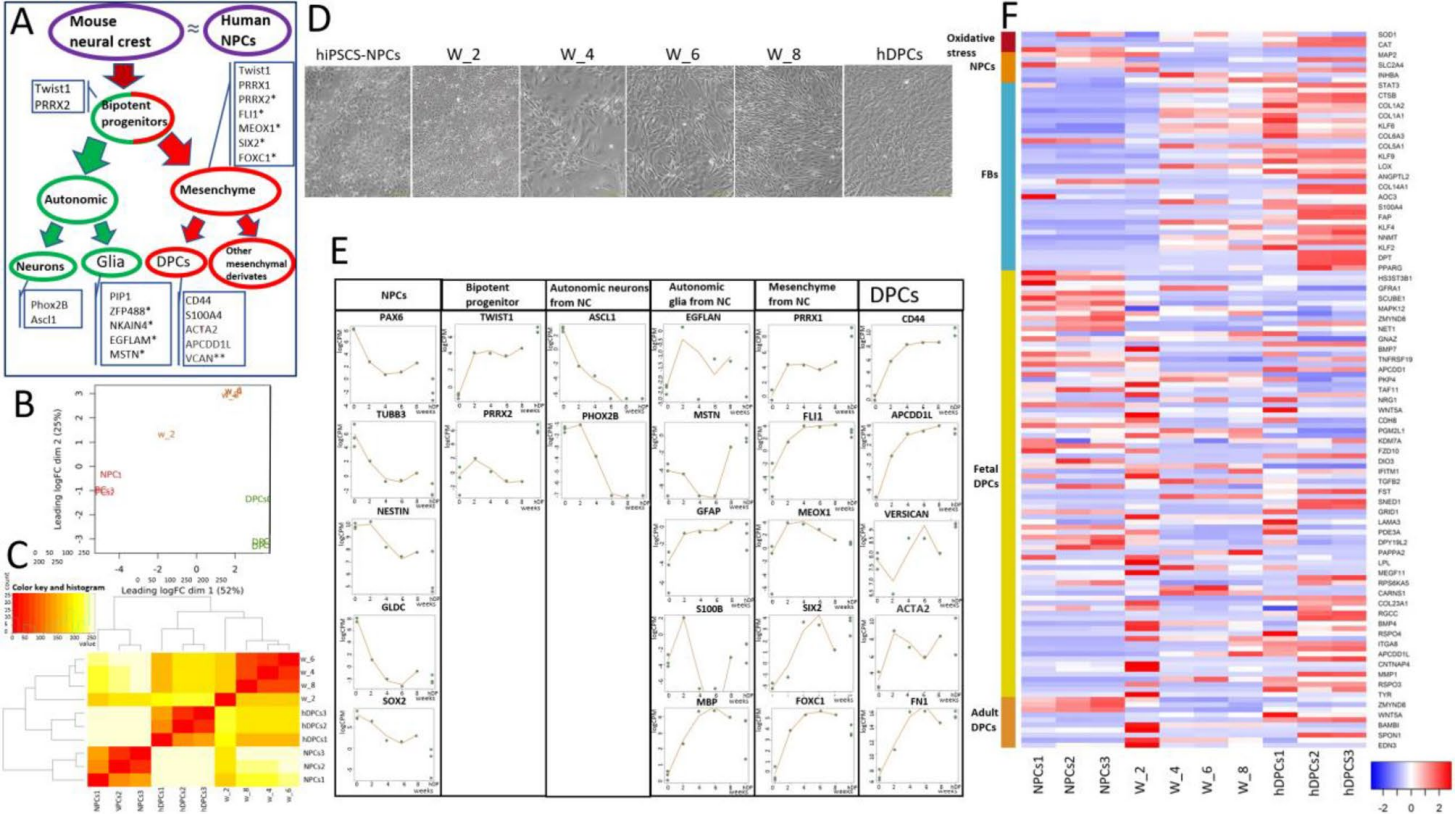
Analysis of bulk hiPSC-DPC RNA-seq data across multiple differentiation time points. (**A**) Schematic showing the differentiation trajectory of NCs, which are facial DPC progenitors, during mouse embryogenesis ^19^. (**B**) Hierarchical clustering heatmap of the Euclidean distance matrix of log_2_(CPM) values for all samples (NPCs 1–3 [negative control], hiPSC-DPCs after 2, 4, 6, and 8 weeks of differentiation, and DPCs 1–3 [positive control]). (**C**) Multidimensional scaling showing the variability between replicates and different treatments in log_2_ fold change (log_2_FC) distance (log_2_FC of the top 500 genes). NPC samples: dark red; DPC samples: green; samples taken weekly: orange. (**D**) Phase contrast images of dermal cell morphology dynamics during hiPSC differentiation. Scale bar: 200 µm (left and right images); 400 µm (center). (**E**) Dot plots for selected gene groups that show the expression profiles of NPCs, bipotent progenitors derived from NCs, autonomic neurons derived from NCs, autonomic glia derived from NCs, mesenchymal stem cells derived from NCs, and DP markers. Dots show the actual expression values for each sample. Lines: values fitted by GLM; *specific markers for unbiased clustering; **some non-specific fibroblast markers with high expression in hDPCs. (**F**) Heatmap illustrating RNA-seq-based DEG expression between NPCs 1–3 (negative control), hiPSC-DPCs after 2, 4, 6, and 8 weeks of differentiation, and hDPCs 1–3 (positive control). The columns indicate samples, and the rows represent up-regulated (red) and down-regulated (blue) genes.

As a positive control in this work, we used hDPCs from the human scalp. In mice and humans, the head mesenchyme develops from NCCs^4^. NCCs give rise to bipotent progenitors, which, in turn, differentiate into glial cells, autonomic neurons, and various populations of mesenchymal stem cells, including DPCs^21^. The phenotype of hiPSCs during the second week of differentiation (W_2) resembled that described by Soldatov et al.^21^: bipotent progenitors of autonomic neurons, glial cells, and mesenchymal progenitors that can differentiate into facial DPCs (Fig. 2A). The similarity between W_2 hiPSC-DPCs and bipotent progenitors was supported by the upregulation of *TWIST1*, *PRRX2*, *PRRX1*, and *PHOX2B* (Fig. 2E). The downregulation of autonomic neuron markers (*ASCL1* and *PHOX2B*) and the upregulation of glial (*EGFLAN*, *MSTN*, *GFAP*) and mesenchymal (*TWIST1*, *PRRX1*, *FLI1*, *SIX2*, *FOXC1*) markers in hiPSCs-NPCs-derived DP-like cells indicates that neural-mesenchymal progenitors differentiate into glial and mesenchymal cells after the second week of differentiation (Fig. 2E). These changes also correlated with morphologic alterations and neuron formation in W_2, which were not observed in hiPSC-NPCs in W_4. The terminal phenotype of hiPSC-DPCs was similar to that of hDPCs (Fig. 2D). Notably, all autonomic glial markers were expressed in both hDPCs and W_8 cells (Fig. 2E). It is possible that some glial subpopulations persist in the embryonic and adult DP niche. The upregulation of highly expressed hDPCs genes (*CD44*, *APCDD1L*, *VCAM*, *ACTA2*, *MFAP5*) in hiPSCs-DPCs, as well as the similar expression levels of conventional mouse bipotent progenitor genes between hiPSC-DPCs and the positive control (hDPCs), can be interpreted as the formation of a DP-like cell subpopulation during W_6 and W_8 of differentiation (Fig. 2E).

We next sought to obtain a more detailed understanding of the cell cycle characteristics of differentiating cells. Therefore, we assessed the dynamics of proliferation marker expression, as well as those of markers of the most common types of programmed cell death: apoptosis, autophagic death, and necroptosis^22–24^ (Figure S2). We observed a decrease in the expression levels of the proliferation markers *MKI67*, *RBBP7*, and *PCNA*, corresponding to the decrease in the proliferation of cultured cells. With respect to apoptotic and autophagic death gene expression, however, these data were not consistent with each other and no unequivocal conclusion could be drawn, because some specific markers of these processes increase and some decrease during differentiation (Figure S2). However, during differentiation, there was a pronounced increase in the expression of the necroptosis markers *RIPK1*, *RIPK3*, and *MLKL*, indicating the potential role of this mechanism in differentiation-associated cell death (Figure S2). This finding also correlates with the important, but not fully understood, role of necroptosis in vertebrate development^25^. The relatively high level of apoptotic, autophagic death, and necroptotic gene expression in hDPCs was further associated with an increase in the frequency of mutations in cells isolated from the skin of adult donors (Figure S2). Also gene ontology (GO) enrichment analysis of the differentially expressed genes was performed (Figure S3).

The aforementioned results also correlate with the temporal gene expression phenotypes of hiPSC-DPCs: W_2 cells differed from the control and other experimental groups, but W_4, W_6, and W_8 cells were identical and more similar to the positive control (hDPCs) than W_2 cells (Fig. 2B,C).

To better understand the phenotypic plasticity of DP-like cells during differentiation and identify the resulting cell population, the expression of neural, fibroblast, and embryonal and mature DPC genes were analyzed. RNA was obtained from hiPSC-NPCs, W_2, W_4, W_6, and W_8 cells, and hDPCs and subjected to bulk RNA-seq analysis. This analysis revealed the downregulation of neural genes (*PAX6*, *TUBB3*, *NESTIN*, *GLDC*, *SOX2*, *MAP2*, and *SLC2A4*) and upregulation of fibroblast genes (*VCAN*, *FN1*, *S100A4*, and *CD44*, among others) in hiPSC-NPCs (negative control) compared to W_8 cells (Fig. 2E,F). Notably, the majority of genes expressed in embryonal and mature DPCs (*BMP4*, *BMP7*, *WNT5A*, *MEGF11*, *LPL*, *COL23A1*, *BAMB1*, and *EDN3*) reached maximal expression levels in W_2 cells (Fig. 2F). The changes in gene expression correlated with the observed morphology conversion (Fig. 2D). Furthermore, with respect to temporal changes in gene expression, W_2 cells differed from the control and remaining experimental groups, but W_4, W_6, and W_8 cells were largely identical and more similar to the positive control cells (hDPCs) than W_2 cells (Fig. 2B,C). Thus, this analysis demonstrates the similarities between mouse NC development and the in vitro differentiation of human NPCs into DP-like cells. The expression patterns of the key markers in DP-like cells (W_8) are similar to those of hDPCs but not identical.

### Co-cultivation of hiPSC-DPCs with KCs and epithelial-mesenchymal interactions

**Figure 3:**
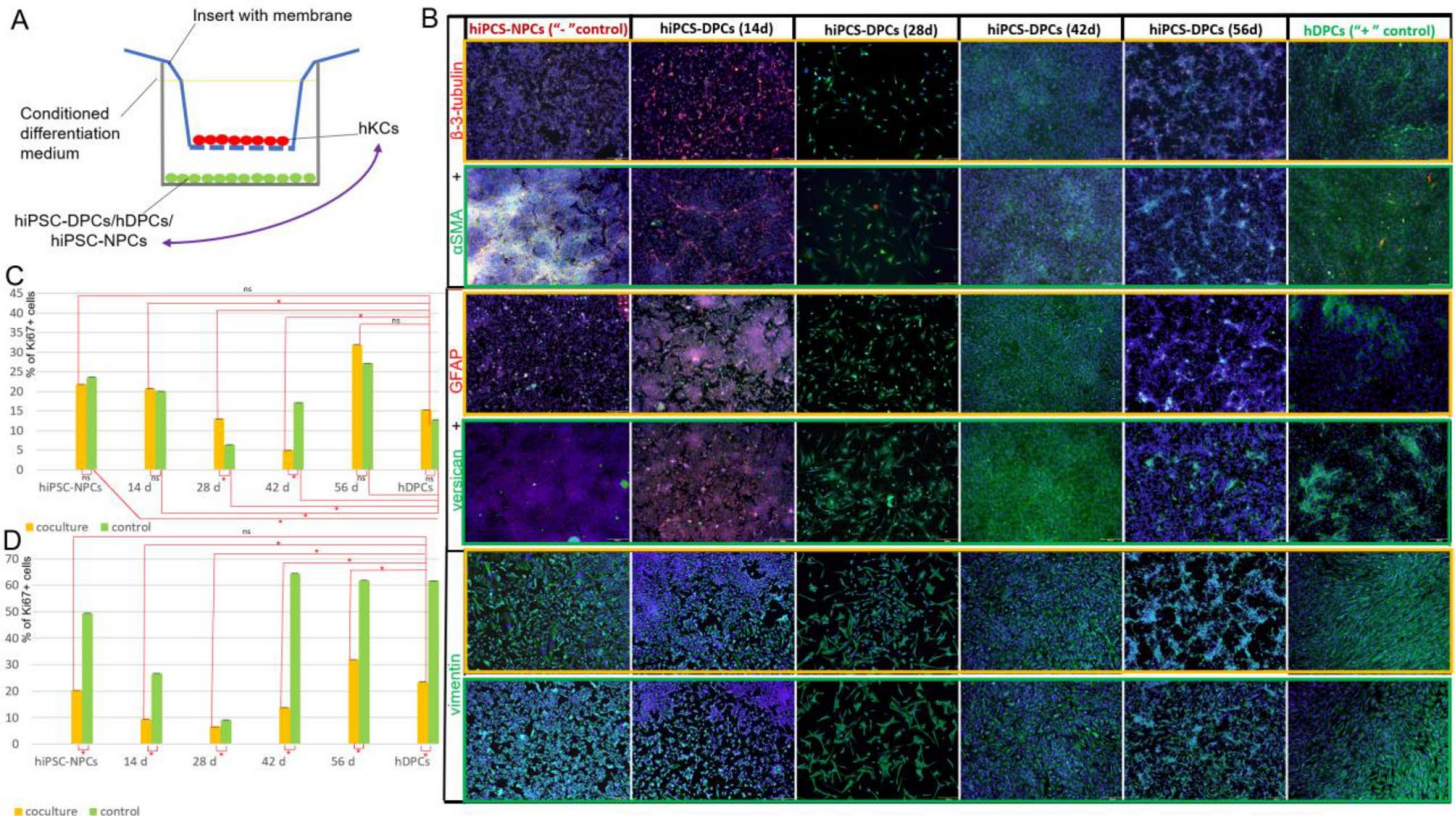
Effects of co-cultivation on hiPSC-DPCs and primary hKCs. (**A**) Schematic showing the differentiation of hiPSCs into DPCs in co-culture. (**B**) Fluorescence-microscopy-based immunohistochemical detection of α-SMA, β-3-tubulin, GFAP, versican, and vimentin in hiPSC-DPCs alone (circled with a green border) and in co-culture with hKCs (circled with a orange border). Scale bar: 200 µm. (**C**) Proportion of proliferating cells during DPC differentiation alone and when co-cultured with hKCs for 4 days. *statistically significant difference; *p* < 0.05. (**D**) Proportion of proliferating hKCs in co-culture during DPC differentiation. * statistically significant difference; *p* < 0.05; ns—non significane; *p* ≥ 0.05.

We previously reported that hiPSC-DPCs are less capable of maintaining folliculogenesis in organoids than hDPCs^26^. To further optimize our differentiation protocol and analyze epithelial-mesenchymal interactions, we evaluated the expression dynamics of neural and specialized marker expression in DP-like cells during differentiation. Another task was to identify the differentiation stage that was most sensitive to interactions with hKCs. Thus, we designed a study with two arms. In the first arm, the cells were differentiated according to our standard protocol. In the second arm, the cells were differentiated in a system with membrane inserts together with hKCs for 4 days (Fig. 3A). We then used immunocytochemistry to assess the expression of proliferation (KI67), neural (β-3-tubulin, GFAP), and dermal cell (versican, α-SMA, and vimentin) markers in control and co-cultivated conditions after 2, 4, and 6 weeks of differentiation. The expression of GFAP, a glial marker, and β-3-tubulin, a marker of immature neurons, were detected only in the negative control and after 2 weeks of differentiation in both the control and co-culture conditions, except for positive β-3-tubulin expression on 56d of differentiation. When DP-like cells were co-cultured with hKCs, neural differentiation was suppressed and the number of β-3-tubulin^+^ cells was significantly lower than in the control. (Fig. 3B; Figure S6). However, unlike the co-cultures, hiPSC-NPCs and hiPSC-DPCs after 2 weeks of differentiation were able to form neurons that expressed β-3-tubulin (Fig. 3B). Dermal differentiation was inducted and the expression of α-SMA after 14d and 28d of differentiation and versican after 14d of differentiation were significantly higher in the coculture compared to the control (Fig. 3B; Figure S6). The expression dynamics of all dermal protein markers changed to the same degree, except for the higher expression of versican observed after 6 weeks of differentiation in the control compared to the co-culture. No other immunohistochemistry-based significant differences were observed (Fig. 3B; Figure S6).

We also analyzed the intensity of proliferation (Fig. 3C). hKCs had a stimulating effect on primary hDPCs cultures. Over the first 14 days, hKCs did not induce the proliferation of differentiating DP cells. On day 28, a significant decrease in the number of cells in the culture was observed (Fig. 3C). At this time point, the neural subpopulations disappeared and the culture behavior in response to co-cultivation with hKCs was similar to that of hDPCs, in that co-cultivation had a significant stimulating effect. However, after 6 weeks of differentiation, hKCs suppressed the proliferation of differentiating cells. Specifically, the effect of co-culture was insignificant in all groups but the percentage of KI67^+^ hKCs was considerably higher than that of hDPCs (Fig. 3C).

Next, the responses of hKCs that were co-cultured with hiPSC-DPCs and hDPCs were analyzed. The effect of co-cultivation on hKCs proliferative activity contrasted with the effect on DP-like cells. Specifically, in co-cultures at all stages of differentiation, there were decreases in the proportions of KI67^+^ hKCs compared to the control (Fig. 3D).

In summary, we conclude that co-cultivation influences hiPSC-DPCs cell morphology and the pattern of β-3-tubulin expression during the second week of differentiation. Beginning at the fourth week of differentiation, the cells retained the expression of dermal markers (α-smooth muscle actin, versican, and vimentin) and did express neural markers (GFAP and β-3-tubulin). However, co-cultivation did not impact the synthesis of these proteins. We also observed that co-cultivation stimulates proliferation in W_4 and W_8 cells and decreases proliferation in W_6 cells and hKCs at all stages of hiPSC-NPCs differentiation. Our data demonstrated that the effects of co-cultivation on differentiated hiPSC-DPCs and hDPCs (positive control) were equal.

### The functional potential of hiPSC-DPCs was analyzed via tubulogenesis, LSE generation, and organoid formation assays

**Figure 4:**
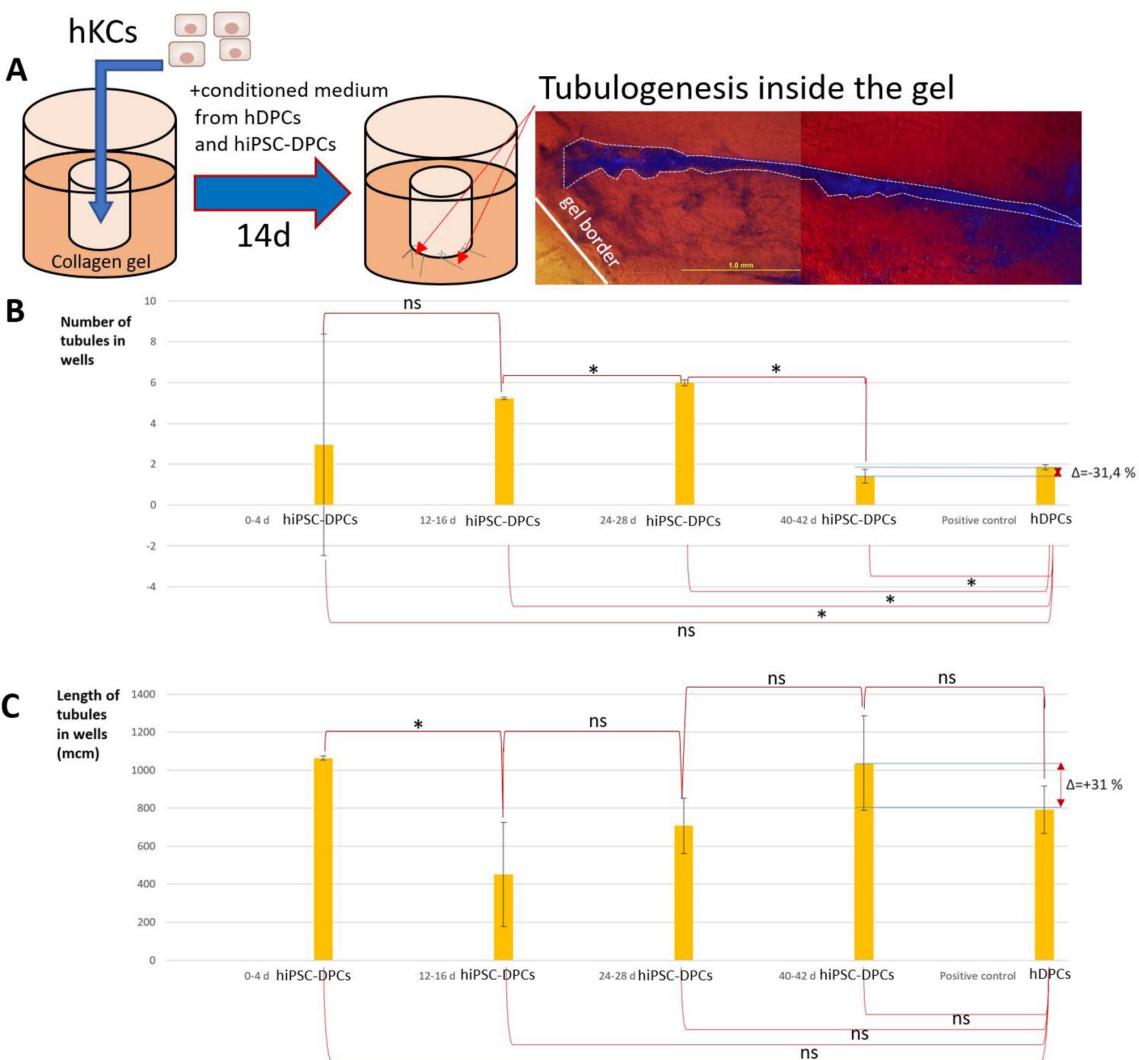
Tubulogenesis assay. (**A**) Schematic showing the tubulogenesis experiment with an example of tubules (bright-field microscopy with DAPI fluorescence). Scale bar: 1 mm. (**B**) Histograms demonstrating the number of tubules in each group: cultivation in medium with 10% conditioned medium from hiPSC-DPCs (0–4 days of differentiation), conditioned medium from hiPSC-DPCs (12–16 days of differentiation), conditioned medium from hiPSC-DPCs (24–28 days of differentiation), conditioned medium from hiPSC-DPCs (40–42 days of differentiation), and conditioned medium from hDPCs (positive control). *statistically significant difference; *p* < 0.05; ns—non significane; *p* ≥ 0.05. (**C**) Histograms demonstrating the tubule lengths in each group: cultivation in medium with 10% conditioned medium from hiPSC-DPCs (0–4 days of differentiation), conditioned medium from hiPSC-DPCs (12–16 days of differentiation), conditioned medium from hiPSC-DPCs (24–28 days of differentiation), conditioned medium from hiPSC-DPCs (40–42 days of differentiation), and conditioned medium from hDPCs (positive control). *statistically significant difference; *p* < 0.05; ns—non significane; *p* ≥ 0.05.

We previously developed a functional assay to evaluate the morphogenetic potential of DPCs by inducing tubular-like outgrowths inside a soft collagen gel in the presence of DP-like cell conditioned medium^12^. Conditioned medium collected from DP-like cells at several differentiation stages was added to KCs embedded within the gel. This treatment enabled KCs to produce tubules differing in number and length (Fig. 4A). Tubulogenesis intensity (the average number of tubules per well) following exposure to conditioned medium from DP-like cells increased from days 0–4 to 24–28 of differentiation, but decreased dramatically at days 40–42 and became similar to that of the positive control. The number of tubules in the positive control was 31.4% higher than that in the group exposed to conditioned medium from differentiated DP-like cells (Fig. 4B).

The greatest average tubule length was observed in the group exposed to conditioned medium from hiPSC-DPCs at the beginning of differentiation and in the group exposed to conditioned medium from hiPSC-DPCs on 40–42 days of differentiation. When medium from cells at day 12–16 was added, tubule length was reduced by more than half. Conditioned medium from DP-like cells obtained led to a two-fold increase in tubule length. Tubule length in the positive control was 31% smaller than that in the group with conditioned medium from fully differentiated DP-like cells (Fig. 4C). Thus, this assay demonstrated that the ability of hiPSC-DPCs to stimulate tubulogenesis is similar to that of control adult hDPCs.These results partially confirmed data in a previous report^12^. Specifically, we showed that conditioned medium from hiPSC-DPCs can modulate the ability of hKCs to form tubules that migrate through a high-density collagen gel, resulting in the generation of different numbers of tubules with different lengths.

To evaluate the ability of hiPSC-DPCs to form three-dimensional aggregates (organoids) in the presence of KCs, suspensions of RFP-labeled hiPSC-DPCs and hKCs were incubated in hanging drop cultures (Figure S4A). Our results were consistent with those of an earlier study of hDPCs and hKCs that demonstrated interactions between these cell types in three-dimensional cultures and recapitulated the early stages of folliculogenesis in vitro^26^. In hanging drops containing KCs and DPCs of various origins, amorphous organoids were formed after 3 days of incubation. We observed that hKCs, hiPSC-DPCs, and hDPCs participated in organoid formation. Expression of versican, a specific DPC marker, was detected immunohistochemically. It was also possible to identify hKCs in organoids using immunohistochemical detection of K6 and K14 (Figure S4B).

The general morphology of the resulting structures was similar to that of hDPCs + hKCs organoids reported previously^26,27^ (Figure S4B). The sizes of DPC-derived structures were identical irrespective of cell origin. However, the morphologies of the organoid epithelial compartments were different. Specifically, while hKCs formed a monolayer on the organoid border with DPCs mimicking the emerging outer root sheath, in organoids obtained using iPSC-DPCs cells, K14 expression was scattered and the formation of a layered structure was not observed (Figure S4B).

To test the ability of hiPSC-DPCs to initiate the early stages of folliculogenesis in vitro, we formed LSEs by incorporating DPCs and hKCs into the wells of high-density collagen gels (Figure S5A,H). Immunohistochemical detection of the basal keratinocyte marker K14 showed successful hKCs integration into systems where hiPSC-DPCs were used as the dermal layer. This analysis showed that hiPSC-DPCs and hKCs, as well as hDPCs and hKCs, could form tubular structures inside collagen wells (Figure S5B,G). Detection of versican and vimentin expression in all of these structures indicated that hiPSC-DPCs participated effectively in morphogenesis. Negative control demonstrates the absence of structures with defined form (Figures S5I,J).

During the formation of LSEs with organoids, hiPSC-DPCs and hDPCs successfully formed an integrated system with hKCs and demonstrated epithelial-mesenchymal interactions. The problems with epidermal stratification and proliferation in dermal layer equivalents (i.e., three-dimensional models of DPCs + KCs) indicate that further optimization is needed^19,28^. A similar protocol that generates hDPCs + hKCs organoids in wells inside of collagen gels yielded better results and enabled the formation of more mature HF-like organoids^13^. These functional phenotypic assays indicate that hiPSC-DPCs can interact with hKCs in three-dimensional cultures and facilitate epithelial cell migration. However, hiPSC-DPCs cannot maintain these epithelial-mesenchymal interactions, impacting the organization of the epithelial compartment of the developing primordium.

## Conclusion

Our hiPSC-NPC dermal differentiation protocol allowed us to obtain a cell line with the characteristics of young fibroblasts that expressed DPC markers and induced early stages of folliculogenesis in vitro. The resulting hiPSC-DPCs interacted with KCs during the formation of organoids in LSEs, initiated tubulogenesis, and integrated into organoids in hanging drop cultures, recapitulating the early stages of folliculogenesis. In addition, W_6 and W_8 cells exhibited expression patterns of fetal and adult DPC markers equivalent to those of adult DPCs. Furthermore, hiPSC-NPCs differentiated into DP-like cells through an intermediate cell type that was similar to the bipotent neural-mesenchymal progenitor observed during mouse NC development. Finally, our data included RNA-seq libraries, which are suitable for the study of the mechanisms and signaling pathways underlying NPC differentiation into dermal cells.

### Supplementary Information

Supplementary Figures.
