# Supplementary material for: Trajectory of hiPSCs derived neural progenitor cells differentiation into dermal papilla-like cells and their characteristics
Source: Sci Rep. 2023 Aug 30;13:14213. doi: 10.1038/s41598-023-40398-w (PMC10469169; doi:10.1038/s41598-023-40398-w)
Supplement: Supplementary file 1 — Supplementary Figures. [file 41598_2023_40398_MOESM1_ESM.docx]

SUPPLEMENTARY INFORMATION

.
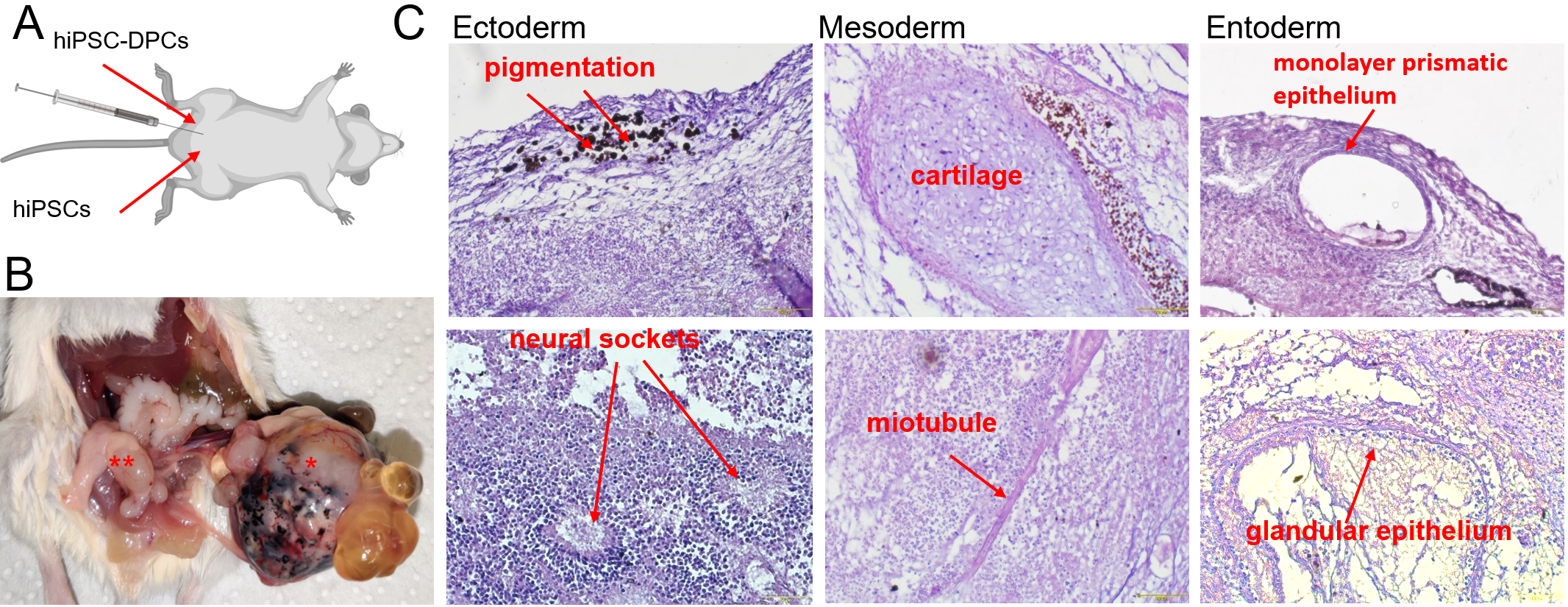


|  |
| --- |
| Figure S1. Tumorigenesis test. A The scheme of tumorigenesis test of hiPSC-DPCs *in vivo.* B General view of animals testicles after the formation of teratomas. * - right testis, where the suspension of hiPSC-DPSc was injected, ** - left testicle, where the hiPSC suspension was injected. C Teratomas sections, demonstrated neoplasms developed from ectoderm, mesoderm and endoderm. Hematoxylin-eosin stain. Scale bar, 100 microns. |


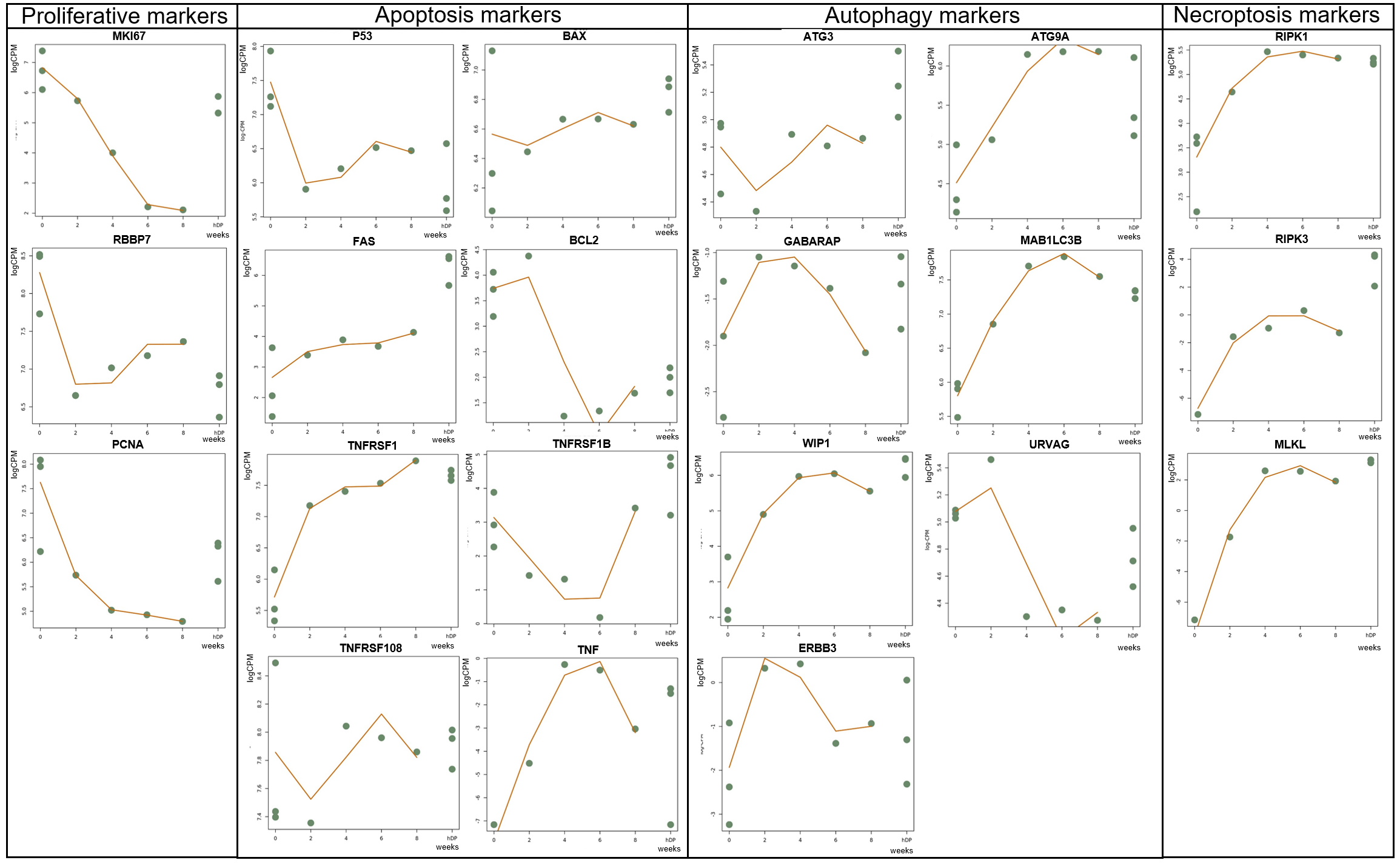


Figure S2. The analysis of bulk RNA-seq data obtained from hiPSC-DPCs at observed timepoints of differentiation, demonstrates the dotplots for selected genes groups showing the expression profiles for proliferation, apoptosis, autophagy and necroptosis markers. Dots show the actual expression values for each sample, lines - values fitted by GLM.


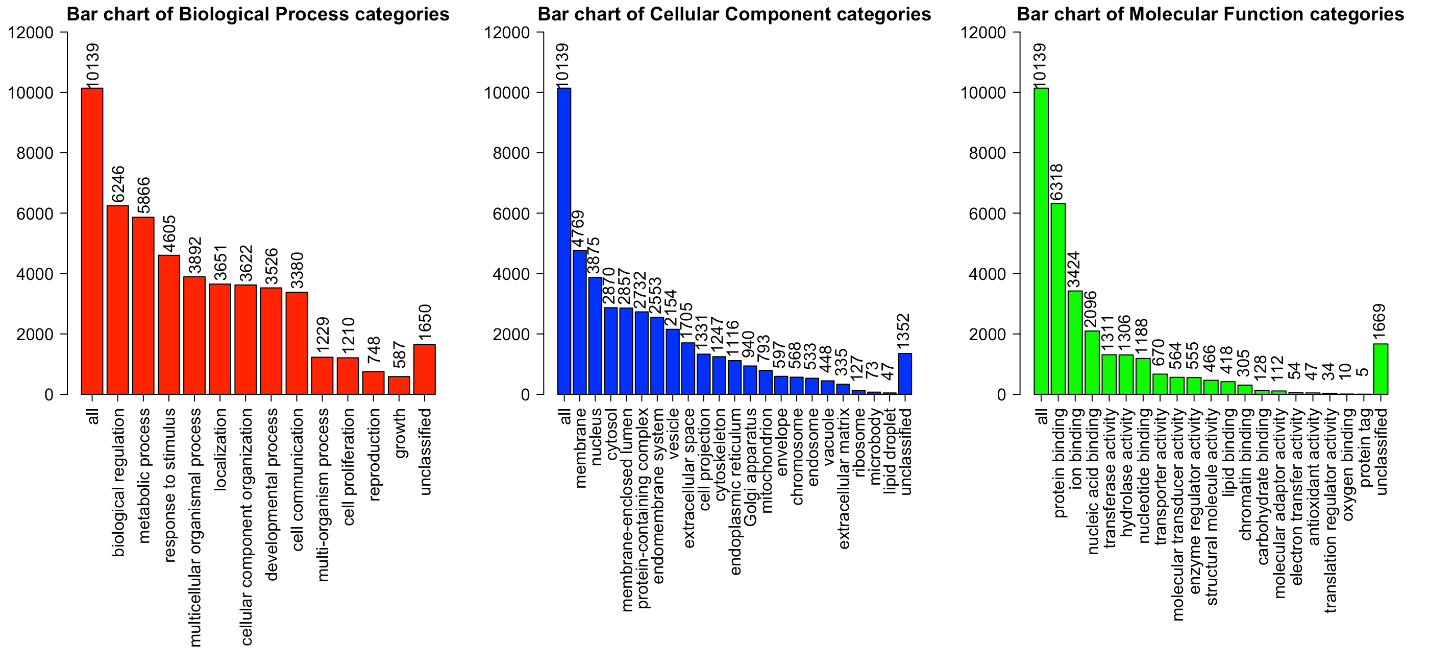
Figure S3. Gene Ontology (GO) enrichment analysis of the differentially expressed genes during hiPSC-NPCs into hiPSCs-DPCs differentiation. Y-scale shows number of genes in each category.


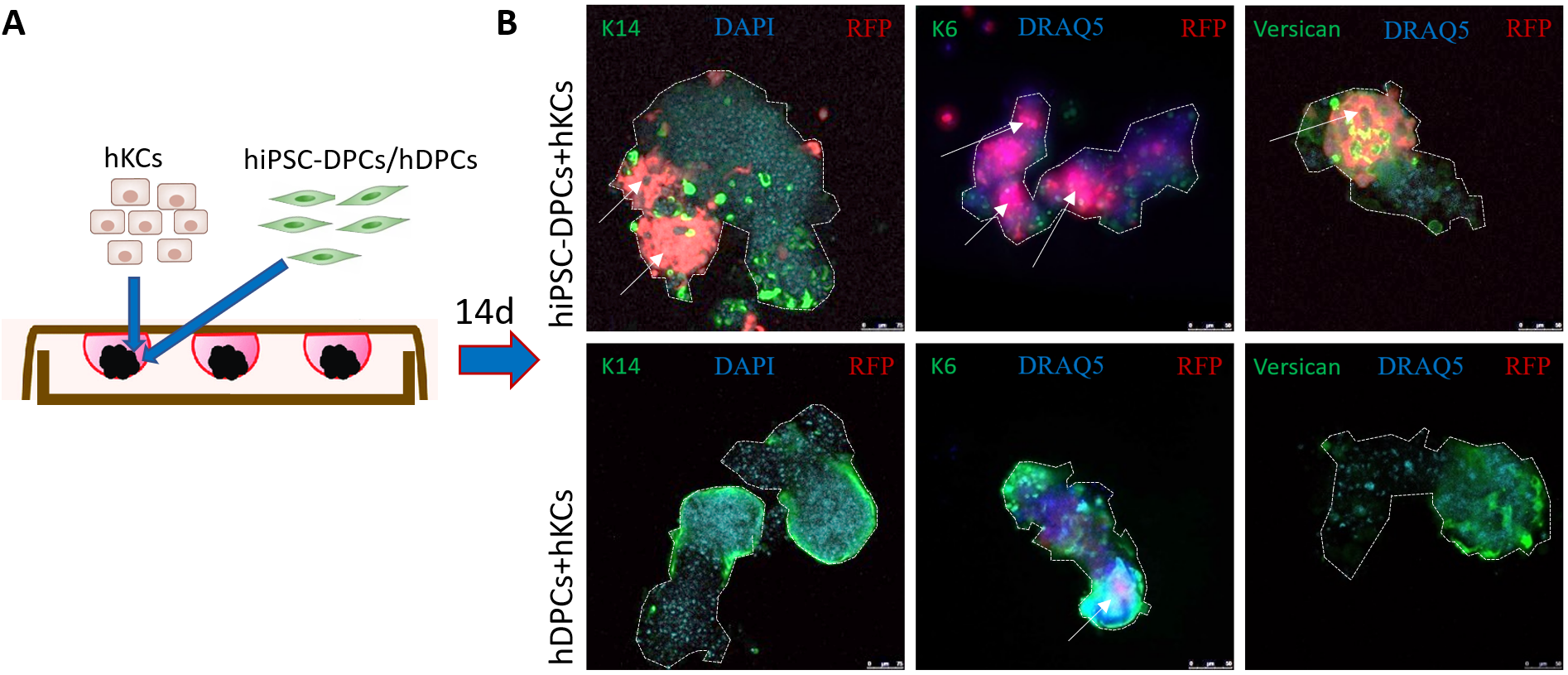


Figure S4. Markers expression in organoids from hDPCs+hKCs and hiPSC-DPCs+hKCs. A The scheme of generation of organoids in hanging drops. B Immunohistochemical detection of K14, K6 and versican in organoids formed by various lines of DPCs and KC. Confocal microscopy. White arrow indicates the clusters DPCs inside the organoids. Scale bar, 50 microns.


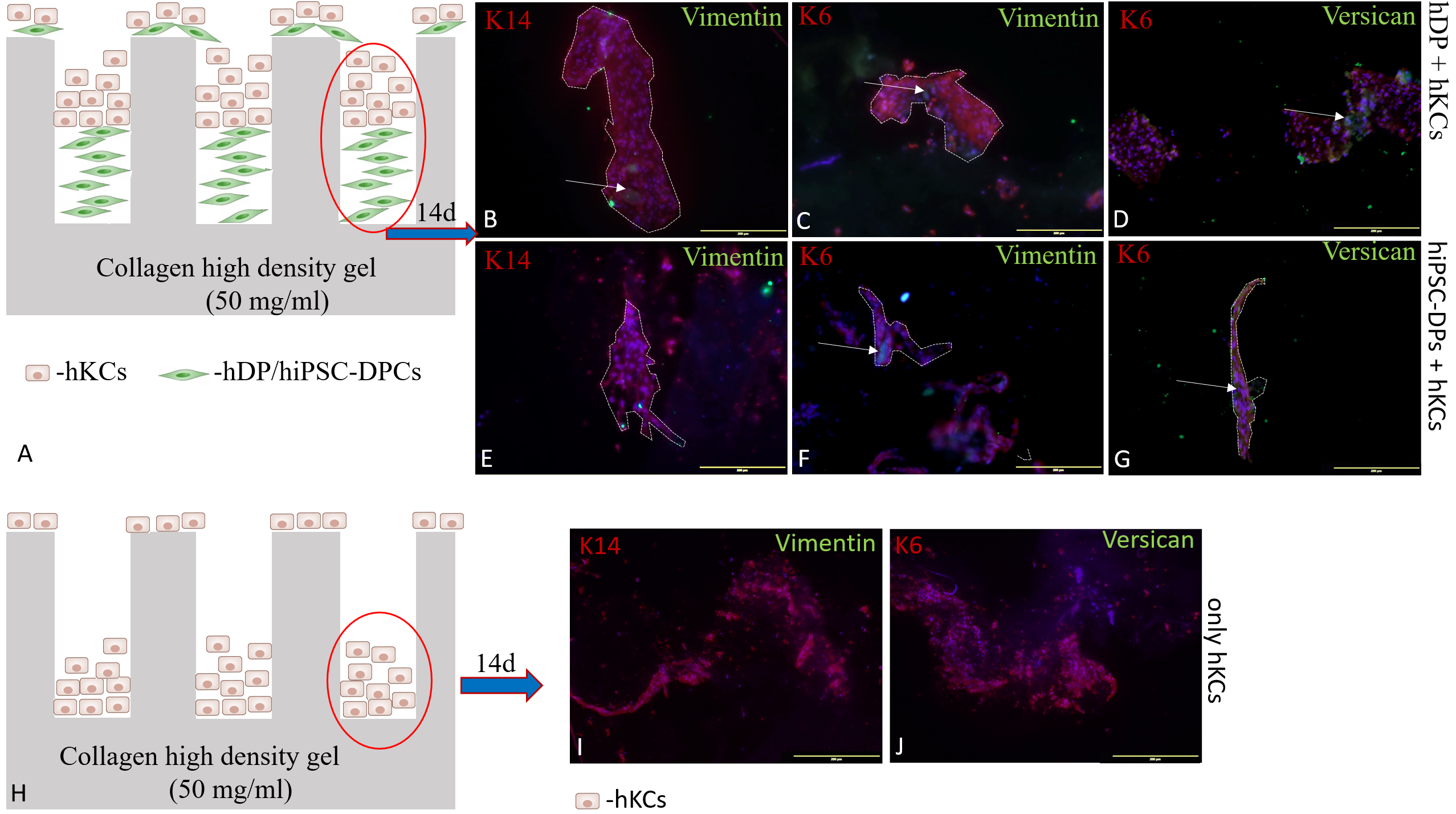


Figure S5. Markers expression in LSEs with integrated organoids on the basis of high-density gel. A The scheme of generation LSEs with integrated organoids, formed by hiPSC-DPCs and hKCs. B-G Immunohistochemical detection of versican, vimentin, K6 and K14 in organoids formed by hiPSC-DPCs and hKCs. The nuclei are stained with DAPI. Scale bar, 100 microns. White arrows indicate clusters of DPCs, expressed vimentin and versican inside the organoids. H The scheme of generation LSEs with integrated organoids, formed by hDPCs and hKCs. I and J Immunohistochemical detection of versican, vimentin, K6 and K14 in organoids formed by hiPSC-DPCs and hKCs. Scale bar, 100 microns.


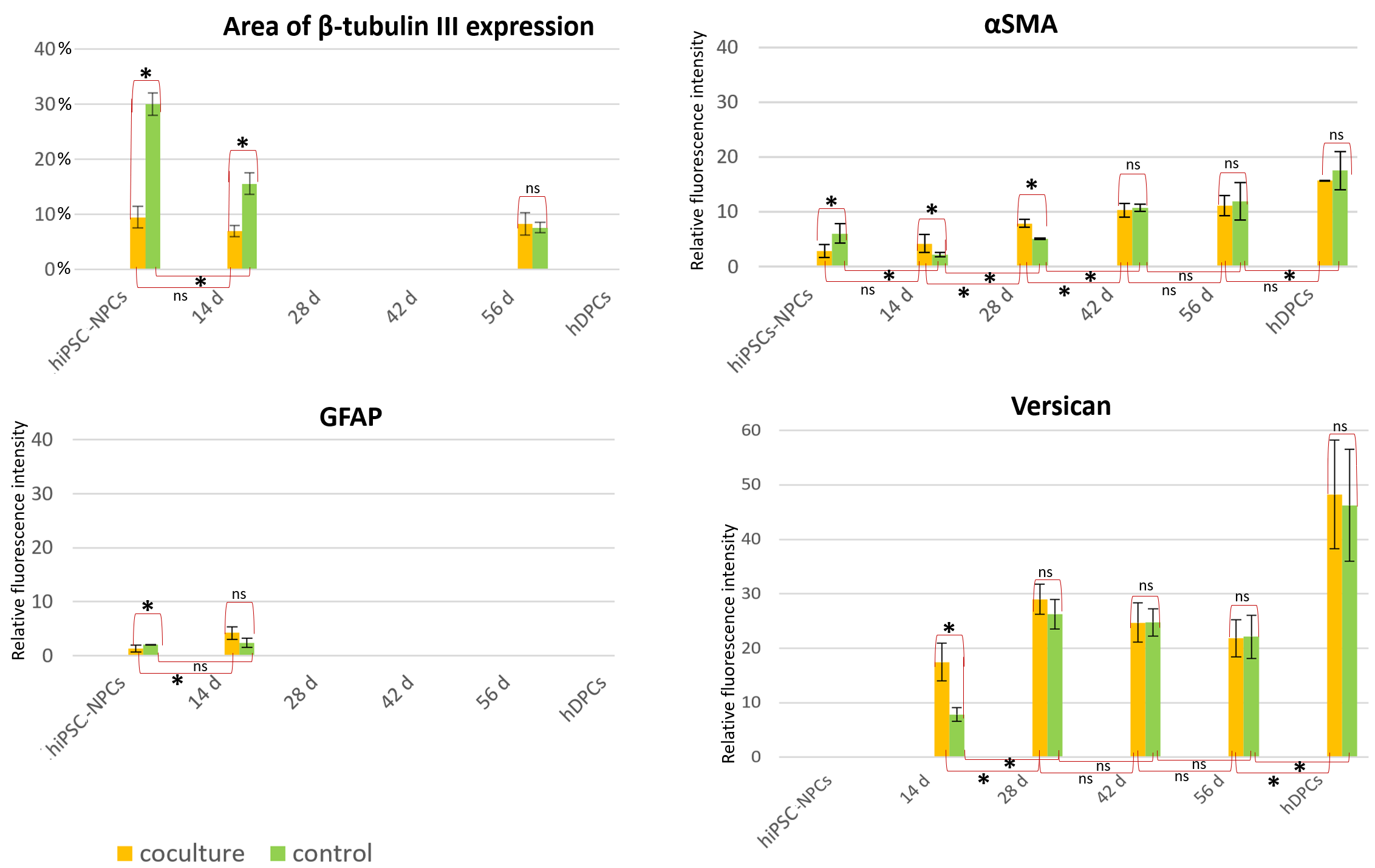


Figure S6. Fluorescence intensity analysis. Dynamic of β-tubulin III area expression and versican, fibronectin and GFAP fluorescence intensity dynamic during hiPSC-NPCs into hiPSC-DPCs differentiation. *p ≤ 0.05, Mann–Whitney test.
